# Clinical and genetic profiles of chinese pediatric patients with catecholaminergic polymorphic ventricular tachycardia

**DOI:** 10.1186/s13023-023-02991-0

**Published:** 2023-12-05

**Authors:** Yu Yan, Liting Tang, Xiaoqin Wang, Kaiyu Zhou, Fan Hu, Hongyu Duan, Xiaoliang Liu, Yimin Hua, Chuan Wang

**Affiliations:** 1 Department of Pediatric Cardiology, West China Second University Hospital, Sichuan University, Chengdu, Sichuan 610041 China; 2 The Cardiac development and early intervention unit, West China Institute of Women and Children’s Health, West China Second University Hospital, Sichuan University, Chengdu, Sichuan 610041 China; 3 Department of Pediatrics, West China Second University Hospital, Sichuan University, No. 20, 3rd section, South Renmin Road, Chengdu, Sichuan 610041 China; 4 West China Medical School of Sichuan University, Chengdu, Sichuan 610041 China; 5 Key Laboratory of Birth Defects and Related Disease of Women and Children (Sichuan University), Ministry of Education Chengdu, Chengdu, Sichuan 610041 China; 6 Key Laboratory of Development and Diseases of Women and Children of Sichuan Province, West China Second University Hospital, Sichuan University, Chengdu, Sichuan 610041 China

**Keywords:** Catecholaminergic Polymorphic ventricular tachycardia, Chinese Pediatric patients, Genetic profiles

## Abstract

**Backgrounds:**

Catecholaminergic polymorphic ventricular tachycardia (CPVT) is a rare but lethal cardiac ion channelopathy. Delayed diagnosis and misdiagnosis remain a matter of concern due to its rarity and insufficient recognition of this disorder, particularly in developing countries like China.

**Aims and methods:**

We reported six catecholaminergic polymorphic ventricular tachycardia (CPVT) children diagnosed in our center along with a comprehensive review of Chinese pediatric CPVT patients reported in domestic and overseas literature between January 2013 and December 2021 to provide an essential reference for physicians to deepen their understanding of pediatric CPVT.

**Results:**

A total of 95 children with CPVT, including our six patients from 21 medical centers were identified. The median age of symptom onset is 8.7 ± 3.0 years. Diagnosis occurred at a median age of 12.9 ± 6.8 years with a delay of 4.3 ± 6.6 years. Selective beta-blockers (Metoprolol and Bisoprolol) were prescribed for 38 patients (56.7%) and 29 (43.3%) patients received non-selective beta-blocker (Propranolol and Nadolol) treatment. Six patients accepted LCSD and seven received ICD implantation at the subsequent therapy. A total of 13 patients died during the disease course. Of the 67 patients with positive gene test results, variants in RYR2 were 47 (70.1%), CASQ2 were 11 (16.4%), and RYR2 accompanied SCN5A were 7 (10.4%). Patients with CASQ2 gene mutations presented with younger symptom onset age, higher positive family history rate and better prognosis than those with RYR2 mutations.

**Conclusion:**

Chinese pediatric patients with CPVT had a poorer prognosis than other cohorts, probably due to delayed/missed diagnosis, non-standard usage of beta-blockers, unavailability of flecainide, and a lower rate of LCSD and ICD implantation.

## Background

Catecholaminergic polymorphic ventricular tachycardia (CPVT) is an inherited arrhythmia disorder in the presence of a structurally normal heart and normal resting electrocardiograph, characterized by adrenergic-induced bidirectional and/or polymorphic ventricular tachycardia (bVT/pVT) in an individual younger than 40 years [1]. Indeed, variants in the ryanodine receptor 2 (RYR2) gene and the calsequestrin (CASQ2) gene are the main contributors to approximately 60–70% of CPVT, leading to excessive calcium leakage from the sarcoplasmic reticulum and delayed afterdepolarizations and arrhythmias. CPVT is a rare but lethal cardiac ion channelopathy, accounting for one of the most common causes of syncope and sudden death in children/adolescents during exercise or emotional stress [2]. Patients with CPVT present for medical attention most frequently around the age of 10 years with exercise-induced syncopal episodes [3]. The mortality of CPVT is up to 31% by the age of 30 years. If left delayed diagnosis, the risk of sudden death and mortality is higher. Hence, early identification and diagnosis should be a significant and vital part of disease treatment.

CPVT has usually presented as case reports as its rarity, leading to insufficient recognition for pediatric doctors, especially in a developing country like China. Therefore, increasing the recognition of pediatric CPVT and reducing delayed and misdiagnosis are valuable for improving prognosis. However, no extensive studies focus on pediatric populations in China. Moreover, CPVT is a gene mutation-caused disease, different mutation types exist in separate people, and hotspot mutation and clinical phenotype may manifest differently. It is vital to make a detailed understanding of the genetic profiles of Chinese pediatric CPVT patients. Using a large cohort of predominately pediatric patients with CPVT, and we sought to investigate the clinical characteristics, genetic profile, and long-term outcomes of CPVT in Chinese children which might present different epidemiology, diagnosis and treatment, as well as prognosis situations compared to other countries. In this study, we reported six CPVT children diagnosed in our center and a comprehensive review of Chinese pediatric CPVT patients reported in domestic and overseas literature to provide an essential reference for physicians to deepen their understanding of pediatric CPVT.

## Methods

We retrospectively reviewed data for children (at < 18 years of age) diagnosed with CPVT between January 2013 and December 2021 at the West China Second University Hospital of Sichuan University (WCSUH-SCU). Written informed consent was obtained from the parents following a full explanation of the nature of the study. The University Ethics Committee on Human Subjects at Sichuan University approved this study.

A pediatric cardiologist confirmed the diagnosis of CPVT by the 2013 h/EHRA/APHRS Expert Consensus Statement on the Diagnosis and Management of Patients with Inherited Primary Arrhythmia Syndromes [1]. The diagnosis criteria include:1) patients in the presence of a structurally normal heart and coronary arteries, normal ECG (Electrocardiogram), and unexplained exercise or catecholamine-induced bVT, polymorphic PVCs or VT in individuals < 40 years of age; 2) patients who have a pathogenic mutation;3) family members of a CPVT index case with an normal heart who manifests exercise-induced PVCs or bVT/pVT; 4) patients with unexplained syncope, unexplained seizures, or sudden cardiac arrest/death. In total, six patients were diagnosed with CPVT. Data regarding clinical features at the time of diagnosis, laboratory parameters, echocardiographic results, treatment, and outcome were systematically collected and analyzed.

Following the 2013 expert consensus recommendations on CPVT therapeutic interventions, patients with CPVT underwent the same treatment program after the diagnosis of CPVT had been established. In patients with CPVT, it suggests changing lifestyle: limit and/or avoid competitive sports, strenuous exercise, and limit exposure to stressful environments. Beta-blockers are recommended in all symptomatic patients with CPVT, and implantable cardioverter-defibrillator (ICD) implantation is recommended in patients with CPVT who experience cardiac arrest, recurrent syncope or polymorphic/bidirectional VT despite optimal medical management, and/or left cardiac sympathetic denervation (LCSD). In addition, the beta-blocker agent is usefully chosen in concealed mutation-positive patients.

To provide a contemporary assessment of variant pathogenicity, all ultra-rare variants (minor allele frequency 0.005 in the Genome Aggregation Database) identified in CPVT-susceptibility genes were reclassified according to the 2015 American College of Medical Genetics and Genomics guidelines [4]. Cascade genetic testing of potentially at-risk relatives was undertaken when appropriate.

In addition, all available literature that described CPVT in Chinese pediatric patients was reviewed via a computerized search. This research was performed with no language restriction via PubMed, Google Scholar and Scopus, by the terms “Bidirectional Tachycardia Induced By Catecholamines or Ventricular Tachycardia, Catecholaminergic Polymorphic, or Catecholamine-induced polymorphic ventricular tachycardia or Catecholaminergic polymorphic ventricular tachycardia or Familial polymorphic ventricular tachycardia or Ventricular Tachycardia, Familial Polymorphic or Ventricular Tachycardia, Familial or Stress-induced polymorphic ventricular tachycardia or Cpvt2 or Ventricular Tachycardia, Catecholaminergic Polymorphic, 2” in Chinese population aged < = 18 years old. The articles written in Chinese were searched using the exact keywords on the China Medical website. An eligible article was included if it reported cases with complete clinical data consistent with the diagnostic criteria of CPVT. The following epidemiologic and clinical variables were evaluated for each patient: demographic data, clinical presentation, diagnostic methods, genetic tests, and outcome. We eliminated the repetition cases reported in the same center.

All data were analyzed using SPSS version 21.0 (SPSS Inc. Chicago, IL, USA). Quantitative data are presented as the mean and range or mean ± standard deviation (SD), while qualitative data are expressed as n/%. The chi-squared/Fisher test and unpaired Student’s t-test were used to compare the differences between the two groups. *P* values < 0.05 were considered statistically significant.

## Results

### Patients’ descriptions in our center

#### Clinical information and diagnosis

**Fig. 1:**
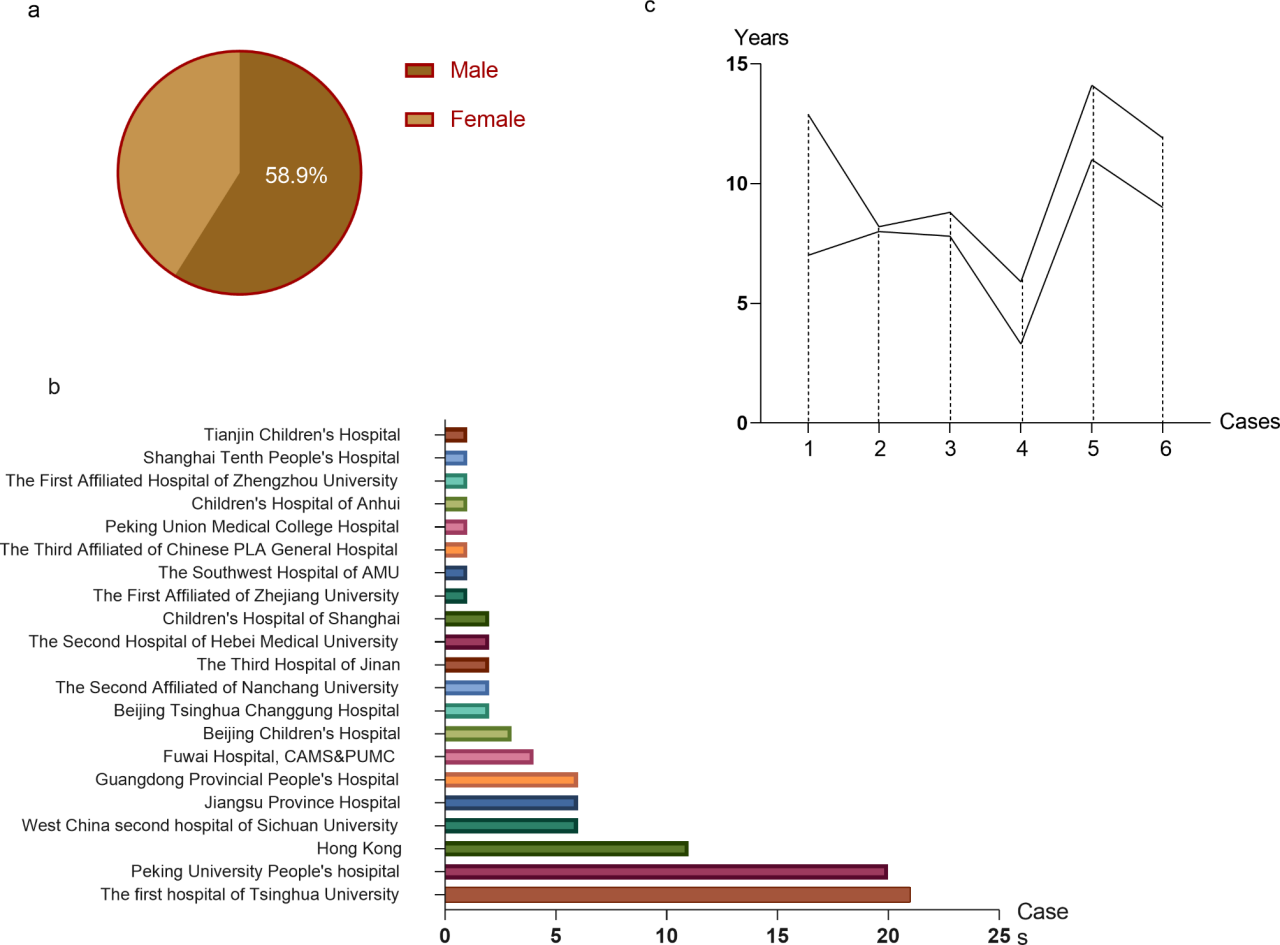
(**a**) Gender distribution of Chinese CPVT patients. Our study included 95 patients with CPVT, 59.4% of them are male. (**b**) Cases reported in Chinese Hospitals. The patients we included are from 21 Chinese pediatric centers. The hospital of Tsinghua University and Peking University People’s Hospital provided the largest samples of 21 and 20 patients, respectively. (**c**) Delayed diagnosis in our center. We reported six cases diagnosed in our center and visually displayed the delay in diagnosis time

Table 1 summarizes six patients with CPVT diagnosed in our center, including five males and one female. They were admitted to our hospital for at least 3 times recurrent syncope, and all their symptoms were triggered by exercise or emotional stress and three patients presented with cardiac arrest during attendance. However, they have no positive family history of sudden cardiac death (SCD), seizure, pregnancy loss, and neonatal death. Two patients first received treatment in neurology and were misdiagnosed with epilepsy, and the other four patients were diagnosed with ventricular arrhythmia and early repolarization syndrome when visiting our department. Diagnosis occurred at a median age of 8.2 ± 3.1 (5.0-11.4) years with a delay to diagnosis of 2.6 ± 2.0 (0.5–4.7) years (Fig. 1c). Sinus bradycardia exists in all patients. In all cases, polymorphic ventricular premature beat (bigeminy and couplets), polymorphic and bidirectional VT with degeneration into ventricular tachycardia was detected during exercise testing. Gene results reported heterozygous mutants of the RYR2 gene in all patients, which is the most frequent variant of CPVT. The classification of RYR2 variant location was based on 4 hotspots: I (amino acids 44–466); II (2246–2534); III(3778–4201); and IV (4497–4959) [5]. RYR2 amino acid was divided into several different domains according to the study by Dhindwal et al. [6] Variant in the NTD domain was found in patient 1 (N-terminal domain, amino acids 1-643), HD1 in patient 2 and patient 4 (amino acids 2110–2679), VSC in patient 3 (amino acids 4594–4719), CTD in patient 5 (C-terminal domain, amino acids 4889–4969), and U-motif in patient 6 (amino acids 4091–4207). Meanwhile, two patients tested more than one potential mutation as SCN5A. Patient 3 and Patient 4 in our cohort were detected with RYR2 mutation accompanied by SCN5A mutation. In Patient 4, a heterozygous mutation of SCN5A, c.677 C > T was reported. Dynamic Electrocardiogram presented multifocal ventricular premature beat, 19 episodes of burst ventricular tachycardia, ventricular bigeminy and QT prolongation but no Brugada pattern in Patient 4. Similarly, a heterozygous mutation of SCN5A, c.3183 A > C emerged in Patient 3. However, pathogenicity analysis reported uncertain in gene result. Prolonged QTc and Brugada patterns were not observed during the therapeutic process.

**Table 1:** Clinical characteristic of six children with CPVT in our center

Patient	1	2	3	4	5	6
Sex	Male	Male	Male	Male	Female	Male
Initial admitted Department	Neurology	Neurology	Cardiology	Cardiology	Cardiology	Cardiology
Primary diagnosis	Epilepsy	Epilepsy	Ventricular arrhythmia	Epilepsy? Ventricular arrhythmia	Ventricular arrhythmia CPVT? Early repolarization syndrome?	Adam-Stokes Syndrome; Ventricular arrhythmia; CPVT?
Age of onset, years	7.0	8.0	7.8	3.3	11.0	9
Age at diagnosis, years	12.9	8.2	8.8	5.9	14.1	11.9
Delay of diagnosis, years	5.9	0.2	1.0	2.6	3.1	2.9
Positive family history	-	-	-	-	-	-
Syncope	Monthly	4 times	7 times	Monthly	3 times	6 times
Cardiac arrest	+	+	-	+	-	-
Trigger						
Exercise	+	-	+	-	+	+
Emotional stress	-	+	-	+	-	-
Genetic results	*RYR2*, c.537T>G	*RYR2*, c.6737 C>T	*RYR2*, c.13952G > T SCN5A, c.3183 A > C	*RYR2*, c.7580T>G SCN5A, c.677 C > T	*RYR2*, c.14876G > A	*RYR2*, c.12284G > A
Positive exercise testing	+	NA	+	NA	+	+
Electrocardiogram	Sinus bradycardia	Sinus bradycardia	Sinus bradycardia	Sinus bradycardia	Sinus bradycardia	Sinus bradycardia
Exercise testing result	Ventricular premature beat and bigeminy	Ventricular premature beat, bVT	pVT, bVT	Ventricular premature beat, pVT	Ventricular premature beat, pVT	pVT, bVT
Medical therapy	Metoprolol	Metoprolol	Metoprolol	Metoprolol	Metoprolol	Metoprolol
Initial dose	0.5 mg/kg/d	0.5 mg/kg/d	0.6 mg/kg/d	0.4 mg/kg/d	1 mg/kg/d	1 mg/kg/d
Maintain dose	1 mg/kg/d	1 mg/kg/d	2 mg/kg/d	1 mg/kg/d	1 mg/kg/d	1.5 mg/kg/d
Beta-blocker failure	-	+	+	-	-	-
Pacemaker	-	-	-	-	-	-
Implantable cardioverter defibrillator (ICD)	-	-	+	-	-	-
Prognosis	Die	Die	Survive	Die	Survive	Survive

#### Treatment and follow up

A summary of the treatment and follow-up is shown in Table 1. In our center, all patients were recommended to avoid strenuous exercise and given long-term beta-blocker (Metoprolol) therapy. No patients received the flecainide treatment due to its unavailability in China. Only patient 3 received ICD therapy and other patients didn’t receive left cardiac sympathetic denervation (LCSD) and ICD implantation due to the refusal by their parents.

In detail, oral Metoprolol was prescribed to Patient 1 (loading dose: 0.5 mg/kg per day; maintenance dose: 1 mg/kg per day). Unfortunately, the patient died suddenly during the morning reading after a 16-months follow-up. The exact dosage of Metoprolol (loading dose: 0.5 mg/kg per day; maintenance dose: 1 mg/kg per day) was prescribed to Patient 2, and he died of emotional stress after discharge for 20 months. For patient 3, oral Propranolol (loading dose: 0.6 mg/kg per day; maintenance dose: 2 mg/kg per day) was taken to him and he received ICD implantation at our center due to recurrent syncope and bVT persisted during the disease course despite on highest tolerated beta-blocker dose. Fortunately, this patient got a successful rescue after the break out of a ventricular arrhythmia because of the ICD placement, and no syncope and/or cardiac arrest was found later. For Patient 4, Metoprolol (loading dose: 0.4 mg/kg per day; maintenance dose: 1 mg/kg per day) was taken under the doctor’s advice. Unfortunately, Patient 4 died from suffocation due to gastric reflux caused by syncope after 33 months of beta-blocker therapy. Patient 5 and Patient 6 were prescribed the same (1 mg/kg/d) Metoprolol dosage in initial medical treatment, and the maintained dosage was 1 mg/kg/d and 1.5 mg/kg/d. Syncope exists with them in occasional attacks after 28- and 18-month follow-ups.

### Patients’ description of the Chinese pediatric cohort

#### Clinical information and diagnosis

To establish the baseline information of Chinese pediatric patients with CPVT, our study included 95 children (59.4% male) with CPVT (Table 2) from 21 Chinese pediatric centers (Fig. 1a). Tsinghua University and Peking University People’s hospital provided the most extensive samples of 21 and 20 patients, respectively (Fig. 1b). Almost all patients (99.0%) were admitted to the hospital for recurrent syncope triggered by exercise or emotional stress and 13 of 34 (38.2%) available patients came up with a cardiac arrest. 16 patients (25.8%) presented with a positive family history of possible CPVT, such as sudden death after exercise of young relatives and parents as the carrier of mutated variants. The median age of Chinese pediatric CPVT patients is 8.7 ± 3.0 (3.0–20.0) years when they present symptomatically. Diagnosis occurred at a median (range) age of 12.9 ± 6.8 (2.1–45.0) years with a delay of 4.3 ± 6.6 (0.0–36.0) years due to the lack of recognition of CPVT. 13 patients received a misdiagnosis of epilepsy. Sinus bradycardia was detected in 20 of 95 (22.1%) patients during ECG and DCG (Dynamic Electrocardiography). 66 of 67 (98.5%) patients performed exercise testing, and 9 presented pVT (22.4%), 11 bVT (18.4%) and 29 (59.2%) had both. Genetic testing was accepted in 79 of 95 (83.1%) patients (NA = 16). Of the 79 patients with genetic test, “negative” genetic results were observed in 12 cases since they only received the gene screening of 45 high mutation exons of RYR2 and CASQ genes and the remaining 60 exons were not screened. In total, genetic test were positive in 67 patients. In detail, variants were RYR2 in 47 of 67 (70.1%), CASQ2 in 11 (16.4%), and RYR2 accompany accompanied SCN5A in 7 (10.4%). Tecrl and LGTs variants were also found in our cohort, and the proportions were 1.4% and 1.4%, respectively.

**Table 2:** The summary of clinical features of Chinese pediatric patients with CPVT

	Patients number, n/N (%)
**Sex, males**	56/95 (58.9)
**Age of onset, years (n = 90)**	8.7 ± 3.0 (3.0–20.0)
**Age at diagnosis, years (n = 92)**	12.9 ± 6.8 (2.1–45.0)
**Delay of diagnosis, years (n = 87)**	4.3 ± 6.6 (0.0–36.0)
**Positive family history (n = 62)**	16/62 (25.8)
**Trigger**	
Exercise, n (%) (n = 53)	41/53 (77.4)
Emotional stress, n (%) (n = 51)	32/51 (62.7)
**Syncope, n (%) (n = 95)**	94/95 (98.9)
**Cardiac arrest, n (%) (n = 34)**	13/34 (38.2)
**Genetic results, n (%) (n = 67)**	
*RYR2*	47/67 (70.1)
*CASQ2*	11/67 (16.4)
*RYR2 + SCN5A*	7/67 (10.4)
*Tecrl*	1/67 (1.4)
*LGTs*	1/67 (1.4)
**RYR2 hotspot, n% (n = 29)**	
I	5/29 (17.2)
II	7/29 (24.1)
III	6/29 (20.7)
IV	8/29 (27.6)
Non-spot	3/29 (10.3)
**RYR2 domain, n% (n = 29)**	
NTD	7/29 (24.1)
SPRY1	1/29 (3.4)
HD1	7/29 (24.1)
Central domain	3/29 (10.3)
U-motif	3/29 (10.3)
VSC	2/29 (6.9)
S6	2/29 (6.9)
CTD	4/29 (13.8)
**Positive exercise testing, n (%) (n = 67)**	66/67 (98.5)
**VT after exercise test, n (%) (n = 49)**	
pVT	9/49(22.4)
bVT	11/49 (18.4)
pVT and bVT	29/49 (59.2)
**Sinus bradycardia, n (%) (n = 95)**	20/95 (22.1)
**First-line therapy**	
**Beta-blocker therapy, n (%) (n = 67)**	
Non-selective	29/67 (43.3)
Selective	38/67 (56.7)
**Beta-blocker therapy in detail, n (%)**	
Nadolol, n (%)	8/67 (11.9)
Metoprolol, n (%)	36/67 (53.7)
Propranolol, n (%)	21/67 (31.3)
Bisoprolol, n (%)	2/67 (3.0)
**Prognosis after beta-blocker therapy, n (%) (n = 47)**	
Remission, n (%)	20/47 (42.5)
Recurrent scope, n (%)	24/47 (51.1)
Death, n (%)	3/47 (6.4)
**Rescue therapy, n (%) (n = 33)**	
Dosage	9/33 (27.3)
Beta-blocker and other drugs, n (%)	10/33 (30.3)
Beta-blocker and LCSD, n (%)	5/33 (15.1)
Beta-blocker and ICD, n (%)	9/33 (27.3)
**Follow-up duration, months (n = 51)**	17.3 ± 23.6
**Pacemaker, n (%)**	4/68 (5.9)
**Outcome, n (%) (n = 75)**	
Remission, n (%)	65/75 (86.7)
Deaths, n (%)	10/75 (13.3)

The data are presented as mean (range) for quantitative variables and as n/% for qualitative data as appropriate

#### Treatment and follow up

Beta-blocker was administrated for most Chinese pediatric patients (100%, NA = 28) as the first-line therapy. Selective beta-blocker was prescribed for 38 patients (56.7%) and 29 (43.3%) patients received non-selective beta-blocker treatment. The medications are as follows: patients initially used Metoprolol in 36 (53.7%), Propranolol in 21 (31.3%), nadolol in 8 (11.9%) and 2 in bisoprolol (3.0%). Chinese doctors prescribed Metoprolol to patients from 0.2 mg/kg/d to 1.7 mg/kg/d with different dosages. Propranolol ranges from 1.1 mg/kg/d to 2.5 mg/kg/d. Nadolol from 10 mg/d to 80 mg/d according to different kilograms. Fewer prescribed Bisoprolol dosages from 2.5 to 5 mg/d. 20 (42.5%) patients got clinical remission (lack of syncope and/or cardiac arrest), and 24 (51.1%) patients still experienced recurrent syncope after beta-blocker therapy. 27.3% of patients were prescribed dosage drugs, 30.3% added other drugs, 6 accepted LCSD and 7 received ICD implantation at the subsequent therapy due to the treatment failure events. The follow-up duration of this cohort was 17.3 ± 23.6 months, and 86.7% (n = 75) of patients obtained clinical remission. 3 patients died in first-line therapy, and 10 died in the whole therapy process.

#### Genetic analysis

Patients’ distribution of RYR2 variant location was 5 in I (17.2%), 7 in II (24.1%),6 in III (20.7%),8 in IV (27.6%) and 3 in non-hotspot (10.3%). 6 patients with the variant in NTD (24.1%), 1 in SPRY1 (amino acids 1084–1217) (3.4%), 7 with HD1 (24.1%), 3 with Central (amino acid 3636–4020) (10.3%), 3 with U-motif (10.3%), 2 with VSC (amino acids 4594–4719) (6.9%), 2 with S6 (amino acids 4836–4888) (2.9%), and 4 with CTD (13.8%). 2 patients died in variant NTD and HD1, respectively. 1 patient died in the variant central domain.

The clinical characteristics of CPVT patients with RYR2 and CASQ2 mutations were compared and the results are shown in Table 3. It was found that the age of symptom onset in patients with RYR2 mutations was older than that in children with CASQ2 mutations (8.3 ± 2.6 years vs. 6.6 ± 1.4 years, *p* = 0.048). Patients with CASQ2 mutations presented a higher rate of positive family history (45.5% vs. 18.2%, *p* = 0.035). In addition, despite no statistical significance, patients with CASQ2 mutations appeared to suffer from a lower rate of cardiac arrest and mortality rate compared to those with RYR2 mutations.

**Table 3:** Clinical comparisons between patients with different gene mutations in pediatric patients with CPVT

	RYR2 mutation (n = 54)	CASQ2 mutation (n = 11)	**P** value
**Sex, males**	32/54 (60.4)	6/11 (50.0)	0.743
**Age of onset, years**	8.3 ± 2.6	6.6 ± 1.4	0.048
**Age at diagnosis, years**	11.0 ± 3.6	10.1 ± 3.7	0.453
**Delay of diagnosis, years**	3.0 ± 3.1	3.4 ± 3.2	0.671
**Positive family history**	8/34 (18.2)	5/11 (45.5)	0.035
**Trigger**			
Exercise	23/30 (76.7)	1/1 (100)	0.750
Emotional stress	19/28 (67.9)	1/1 (100)	0.357
**Syncope**	54/54 (100)	11/11 (100)	/
**Cardiac arrest**	8/17 (56.3)	0/3 (0)	0.145
**Positive exercise testing**	39/39 (100)	7/7 (100)	0.309
**Ventricular arrhythmia**	25/25 (100)	5/5 (100)	/
**Sinus bradycardia**	15/54 (28.3)	1/11 (9.1)	0.179
**Beta-blocker therapy**			0.301
Non-selective	19/38 (50.0)	3/9 (33.3)	
Selective	19/38 (50.0)	6/9 (66.7)	
**Pacemaker**	2/39 (5.1)	0/9 (0)	0.657
**ICD**	5/39 (10.2)	1/9 (11.1)	0.688
**Deaths**	9/36 (23.1)	1/10 (9.1)	0.375

The data are presented as mean (range) for quantitative variables and as n/% for qualitative data as appropriate

## Discussion

In the present study, we first described the clinical features of Chinese pediatric CPVT patients. Several important issues were uncovered and deserved to be emphasized. Firstly, due to the limited recognition and low awareness, a delayed or missed diagnosis of pediatric CPVT is more common and concerning in China. Secondly, the overall mortality rate is relatively high in the Chinese pediatric population, which may result from delayed/missed diagnosis, non-standard usage of beta-blockers, unavailability of flecainide, and a lower rate of LCSD and ICD implantation. Lastly, compared to other abroad cohorts, CASQ2 mutation is more common in Chinese pediatric CPVT patients, and these children presented with a relatively lower trend in onset age, higher rate of positive family history, and better prognosis in comparison with those with RYR2 mutation. These findings were beneficial in highlighting the limitations in China, assisting in clinician awareness of this disorder as well as making the most optimal decision in treatment.

Overall, several points may contribute to and explain the high mortality rate (13.3%) in the Chinese pediatric CPVT cohort. First, owing to the low recognition of this disorder in general, the median delayed duration from disease onset to diagnosis in our center and the whole Chinese cohort were 2.6 ± 2.0 years (0.5–4.7 years) and 4.3 ± 6.6 years (0.0–36.0 years), which were much longer than that in other cohorts [7]. Despite the unremarkable baseline ECG present in CPVT patients, some features may help pediatricians to identify individuals. Most CPVT patients demonstrate a prominent sinus bradycardia in resting ECG [8], which may result from the diastolic calcium leakage from the ryanodine receptor, resulting from either RyR2 or CASQ2 mutations. Indeed, all patients diagnosed in our center had sinus bradycardia and 20 of 95 (22.1%) patients in the whole cohort also came up with varying degrees of sinus bradycardia. Therefore, CPVT should be highly suspected in young patients with sinus bradycardia and syncope/cardiac arrest induced by exercise or emotional stress, which could lead to a timely diagnosis and optimal management. Second, Beta-blocker, ideally non-selective (nadolol or Propranolol), are recommended in all CPVT patients since selective Beta-blocker were proved to be associated with a higher risk of life-threatening arrhythmic events [9, 10]. However, it was found that 56.7% of CPVT cases in our cohort (including the whole six patients in our center) received the selective beta-blocker (Metoprolol and bisoprolol) as the initial choice. Moreover, the issue of sub-therapeutic dosing was also common in most patients. Third, flecainide could reduce ventricular arrhythmias in patients with genotype RYR2-positive CPVT [11]. However, few patients were prescribed flecainide in our cohort due to the unavailability in mainland China. Last, LCSD is an effective anti-fibrillatory intervention for patients with CPVT [12] and ICD implantation was associated with reduced mortality in high-risk CPVT cases [13, 14]. However, only nine and five children received ICD implantation and LCSD surgery, respectively.

Genetic testing is significant and helpful not only for CPVT diagnosis confirmation but also for risk stratification. In the whole Chinese cohort, it was consistent with previous studies [15] that RYR2 variants were identified in most of the CPVT cases (68.4%). However, patient with CASQ2 mutations seems more common in Chinese CPVT patients (16.9%) than in another cohort (4.6%) [16]. Compared with patients with RYR2 mutations, those with CASQ2 mutations presented with an earlier age of disease onset, more positive family history as well as fewer malignant cardiac events, which was consistent with previous studies [17, 18]. Andrea Mazzanti reported in 2022 [13] that patients with RYR2 variants affecting the C-terminal domain (CTD, amino acids 4889–4969) were at higher risk of β-blocker failure, independently of clinical presentation and β-blocker type used. Unfortunately, the role of RYR2 variant hotspots and domains in predicting β-blocker treatment effectiveness as well as adverse cardiac events could not be investigated in our Chinese cohort due to the unavailability of genetic information in most cases and needs to be further explored and verified.

In spite of the progress in genetic testing for CPVT, several issues remain to be determined. First, although several other genes, including TECRL, TRDN, CALM1-3, SCN5A and KCNJ2 apart from RYR2 and CASQ2 have been reported to be associated with CPVT [19]–[21], negative genetic testing is also observed in some cases and other underlying genes and/or variants warrant to be further uncovered. In addition, the negative genetic testing may also be related to gene test methods (gene panel, GWES or GWAS), sequencing depth as well as variant classification criteria. Therefore, it should be emphasized that genetic testing should not be used to rule out the diagnosis when clinical suspicion exists. Second, some of the negative genetic testing patients may have variants of unknown significance (VUS) and the classification of VUS indeed remains a current challenge in the genetic field. Assigning erroneous classifications to variants carries great danger, both for false positives (assigning pathogenic causality to variants that are not) that can have severe consequences, for example leading to the implantation of an unnecessary ICD or, on the contrary, leaving as VUS variants those that are genuinely causative of the disease. Therefore, clinical translation of VUS should be undertaken with caution and should not be excluded or used in clinical decision-making until follow-up testing is completed and its clinical role clarified.

Our study also has some limitations. Our data were extracted from published literature and we couldn’t get more valuable information, such as gene tests and exercise stress tests of patients, to conclude precise results. However, our review still firstly makes the construction of Chinese pediatric CPVT patients.

## Conclusion

Our study first analyzed the Chinese pediatric CPVT patients, providing that Chinese CPVT children presented with longer delayed diagnosis and higher death rates compared to foreign cohorts due to the insufficient recognition of this disorder, the non-standard use of beta-blockers, unavailability of flecainide as well as lower rate of LCSD and ICD implantation. Genetic profiles in Chinese pediatric patients differ from other cohorts: CASQ2 mutation is more common in China and was found to have a relatively lower trend in onset age and mild symptoms in the clinic. This provides an essential reference for clinicians to improve the diagnosis and treatment of CPVT in Chinese children. Heightening the awareness of disease mechanisms, clinical manifestation, differential diagnosis, and developing mechanic therapeutics might help to improve the prognosis and reduce mortality.

## Data Availability

The datasets generated and/or analyzed during the current study are available in the [PubMed, Google Scholar and Scopus] repository, [pubmed.ncbi.nlm.nih.gov, scholar.google.com, www.scopus.com], and the datasets used and/or analyzed during the current study are available from the corresponding author on reasonable request.

## Abbreviations

CPVT: Catecholaminergic polymorphic ventricular tachycardia
bVT: Bidirectional ventricular tachycardia
pVT: Polymorphic ventricular tachycardia
RYR2: Ryanodine receptor 2
CASQ2: Calsequestrin
ECG: Electrocardiogram
DCG: Dynamic cardiogram
ICD: Implantable cardioverter-defibrillator
LCSD: Left cardiac sympathetic denervation
SCD: Sudden cardiac death
BrS: Brugada syndrome
LQTs: Long QT syndrome
SAN: Sinoatrial node
AF: Atrial fibrillation
SSS: Sick sinus syndrome PCCD Progressive cardiac conduction defect
MEPPC: Multifocal ectopic Purkinje-related premature contraction
SID: Sudden infant death syndrome
DCM: Dilated cardiomyopathy
ARVC: Arrhythmogenic right ventricular cardiomyopathy
